# Retraction: MicroRNA-143 Targets Syndecan-1 to Repress Cell Growth in Melanoma

**DOI:** 10.1371/journal.pone.0205767

**Published:** 2018-10-10

**Authors:** The PLOS ONE Editors

After publication of this article [1], concerns were raised about similarities between FACS plots shown in Figs 2E, 2F, and 5D:

- In Fig 2E and 2F, the pre-scramble, anti-scramble, and anti-miR-143 panels contain clusters of data points that appear similar.
- In Fig 2E and Fig 5D, the pre-scramble+NC siRNA, both pre-miR-143, and the Syn-1 siRNA panels contain clusters of data points that appear similar.

We discussed these concerns with the authors who noted that the original data underlying these figures were no longer available. They provided supporting figures with apoptosis and proliferation data from different types of experiments but did not provide the raw data underlying the new results and were unable to clarify the issues about the published FACS data.

We consulted members of *PLOS ONE*'s Editorial Board about this article and about the new data provided post-publication. They advised that the published FACS plots showing similarities appear to have been derived from the same file using different gating parameters, and they further raised that experiments in the article used normal skin rather than isolated melanocytes as control samples to compare against melanoma samples, hence conclusions pertaining to melanoma were not supported. Overall, based on the advice we received, the concerns raised call into question the validity of the conclusions and whether they are sufficiently supported by the reported data.

The authors did not respond to queries regarding the availability of raw data underlying the other results reported in the published article.

In light of these concerns, the *PLOS ONE* Editors retract this article.

The authors did not comment on the retraction decision.
